# Are extended and continuous infusions of time dependent antibiotics used in the management of sepsis in england today?

**DOI:** 10.1186/2197-425X-3-S1-A396

**Published:** 2015-10-01

**Authors:** JA Dunning, JC Roberts

**Affiliations:** Salford Royal Hospital, Undergraduate, Manchester, United Kingdom; Intensive Care / Anaesthesia, Blackburn Royal Hospital, Blackburn, United Kingdom

## Introduction

Sepsis remains a leading cause of death in intensive care units. Administration of appropriate antibiotics within the first hour of diagnosis has been found to be the most effective intervention associated with a reduction in mortality [[1]]. Currently pharmacokinetic profiles of antibiotics are determined by phase II clinical trials that are undertaken by fit and well volunteers however many studies have shown that this data cannot be extrapolated accurately to the critically unwell [[2]]. Some have proposed that continuous and extended infusions of time dependent antibiotics maximise efficacy by increasing the time in which serum concentration is maintained above the minimum inhibitory concentration therefore improving clinical outcomes.

## Objectives

This survey aimed to determine how many intensive care units in England currently use extended and continuous infusions of time dependent antibiotics in the treatment of sepsis.

## Methods

One ICU in each acute trust in England was contacted via telephone in June 2014. A doctor working on the unit grade CT1 or above was then interviewed about antibiotic prescribing in sepsis.

## Results

Of the 148 acute trusts with critical care facilities in mainland England 123 replied to the survey, 23 were unable to be contacted and 2 declined to respond; a response rate of 83.1% was achieved. Of the trusts that responded antibiotic infusions were never used as a clinical strategy in the management of septic patients in 66 (54%). Of the 57 (46%) trusts that did consider using antibiotic infusions for the management of septic patients only 11 (7%) used both continuous and extended infusions of antibiotics, 27 (18%) used continuous infusions only and 19 (15%) used extended infusions but not continuous infusions (Figure 1). Of the 57 trusts that used infusions of antibiotics; Vancomycin was administered by infusion in 48 (84%) trusts and beta-lactams in 16 (28%).

## Conclusions

There remains disagreement regarding the clinical benefit of continuous and extended infusions of time dependent antibiotics in the management of septic patients in ICU. This is despite a large body of theoretical evidence based on pharmacokinetic studies. We suggest further, larger trials powered towards mortality are indicated for consensus to be achieved.

## References

[1] Díaz-Martín A, Martínez-González ML, Ferrer R, Ortiz-Leyba C, Piacentini E, Lopez-Pueyo MJ, Martín-Loeches I, Levy MM, Artigas A, Garnacho-Montero J (2012). Antibiotic prescription patterns in the empiric therapy of severe sepsis: combination of antimicrobials with different mechanisms of action reduces mortality. Critical Care.

[2] Blot S, Lipman J, Roberts DM, Roberts JA (2014). The influence of acute kidney injury on antimicrobial dosing in critically ill patients: are dose reductions always necessary?. Diagn Microbiol Infect Dis.

